# Luminescent metal clusters/barium sulfate composites for white light-emitting devices and anti-counterfeiting labels†

**DOI:** 10.1039/c7ra11804j

**Published:** 2018-01-15

**Authors:** Min Wang, Zhenzhen Huang, Zilong Guo, Wensheng Yang

**Affiliations:** State Key Laboratory for Inorganic Synthesis and Preparative Chemistry, College of Chemistry, Jilin University Changchun 130012 P. R. China wsyang@jlu.edu.cn +86-431-85168086 +86-431-85168185

## Abstract

The applications of luminescent metal nanoclusters (NCs) in device fabrication have been greatly limited by their tough solidifying process and poor stability. In this work, we report the facile preparation of metal NCs/barium sulfate (BaSO_4_) composites by incorporating luminescent metal NCs into BaSO_4_ matrix by subsequent addition of Ba^2+^ and SO_4_^2−^ ions into the aqueous dispersions of the metal NCs. The resulting NCs/BaSO_4_ composites maintained the luminescent colour of the metal NCs and possessed improved stability under external stimuli, such as heating, strong acid and organic solvents. The solid metal NCs/BaSO_4_ composites with blue, green and red emissions were suitable for the fabrication of white light-emitting devices (WLEDs) and multicolour anti-counterfeiting labels.

## Abstract 2

Metal NCs with blue, green and red emissions were incorporated into barium sulfate matrix to improve their stability and performance.

## Graphical abstract

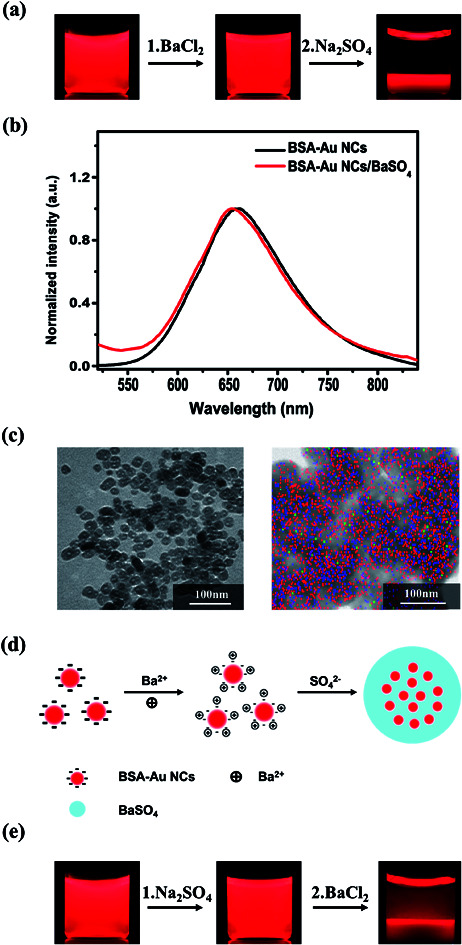

## 1. Introduction

Metal nanoclusters (NCs) consisting of several to tens of metal atoms (Au, Ag or Cu) have emerged as one of the most intriguing materials owing to their unique electrical and optical properties and application potentials in sensing, imaging and lighting techniques.^1–4^ During the preparation of metal NCs, usually organic molecules containing thiol groups or biomolecules are employed as capping agents to direct the formation of the NCs.^5,6^ On the one hand, the “soft” nature of these capping agents endow the NCs with excellent water solubility and good biocompatibility, benefiting their applications in biolabeling and bioimaging.^7–9^ On the other hand, the “soft” nature makes the NCs hard to be transferred into controllable solid-state and readily to undergo precipitation, aggregation and/or oxidation under a variety of external stimuli such as acid, base, heavy metal ion, heating, *etc.*, diminishing their potentials in monolithic applications such as anti-counterfeiting, surface patterning and light emitting device fabrication.^10–13^

During the past decade, considerable efforts have been devoted to address these inherent issues of metal NCs. *In situ* synthesis of luminescent metal NCs embedded in solid template, such as glass and zeolite, is one of the effective ways to solidify and improve the stability of the metal NCs. Qiu *et al.* reported the preparation of luminescent Ag NCs inside a glass through femtosecond laser induced multiphoton reduction of Ag^+^ ions followed by thermal treatment.^14^ Vosch *et al.* developed an approach for synthesis of stable Ag NCs in zeolite by an ion-exchange process and successive heat treatment.^15^ Such *in situ* approach is limited by the reduction potential of metal ions and ion-exchange capacity of the solid template. An alternative way to solidify and improve the stability of the NCs is to incorporate the pre-prepared metal NCs into solid matrix. Yan *et al.* localize glutathione-capped Au NCs on the surface of layered double hydroxides nanosheets *via* layer-by-layer assembly.^16^ Wang *et al.* reported the incorporation of glutathione-capped Au NCs and BSA-capped Cu NCs into silica matrix with the assistance of polyelectrolyte.^17^ Usually these approaches involve cost and/or time-consuming reaction/purification procedure, which hinders the large-scale preparation of the solid-state materials. In addition, due to the limited durability of the solid matrix itself, the NCs composites are less stable in some harsh conditions, such as high temperature and strong acidic/basic medium.

As one of the relatively stable inorganic materials, barium sulfate (BaSO_4_), commonly referred to as barite, is suitable for many diverse uses because of its high specific gravity, opaqueness to X-ray, inertness and whiteness.^18^ For example, it has been used as filler and additive in polymers and paints, catalyst carriers and reflector material of optical devices.^19,20^ Recently, luminescent CdTe quantum dots and carbon nanodots have been incorporated into BaSO_4_ matrix to form solid-state composites with excellent stability.^21,22^

Here we presented the first report to solidify the metal NCs by using BaSO_4_ as matrix. Both organic ligand and biomolecules-capped metal NCs could be incorporated into BaSO_4_ matrix effectively by subsequent addition of barium chloride and sodium sulfate into the dispersions of the NCs. The entire preparation was achieved in 10 min at room temperature without requirement for the addition of extra templates or organic solvents. The resulting solid-state metal NCs/BaSO_4_ composites maintained the luminescent colours of the original NCs and possessed high stability under external stimuli, such as heating, strong acid and organic solvents. White light-emitting devices and anti-counterfeiting labels were readily fabricated by using the NCs/BaSO_4_ particles with red, green and blue emissions.

## 2. Experimental section

### 2.1. Reagents and materials

Bovine serum albumin (BSA), l-glutathione in the reduced form (GSH) and sodium hydroxide (NaOH) were obtained from Sigma-Aldrich. Hydrogen tetrachloroaurate trihydrate (HAuCl_4_·3H_2_O) were provided by Sinopharm Chemical Reagent Co., Ltd. Ethanol (EtOH), methanol (MeOH), and acetonitrile (ACN) were purchased from Aladdin Industrial Corporation. Copper(ii) sulfate pentahydrate (CuSO_4_·5H_2_O), barium chloride (BaCl_2_), sodium sulfate (Na_2_SO_4_) and hydrochloric acid (HCl) were purchased from Beijing Chemical Works. Tetrakis(hydroxymethyl)phosphonium chloride (THPC) and 11-mercaptoundecanoic acid (11-MUA) were purchased from Energy Chemical. Silicone resin OE-6630A and OE-6630B were purchased from Dow Corning Co., Ltd. GaN LED chips without phosphor coating were purchased from Advanced Optoelectronic Technology Co., Ltd. All chemicals were analytical grade and used without further purification. Ultrapure water with a resistivity of 18.2 MΩ cm^−1^ was used as the general solvent throughout the experiments. The glassware were cleaned in a bath of freshly prepared aqua regia (HCl : HNO_3_, 3 : 1 by volume) and rinsed thoroughly in water prior to use.

### 2.2. Instrumentation

Photoluminescence spectra were recorded on a Shimadzu RF-5301PC spectrofluorophotometer. Transmission electron microscopy (TEM) observations were performed on a JEOL-2010 microscope operating at 100 kV. Zeta-potential measurements were obtained with a Brookhaven 90Plus Zeta laser light scattering system. Element analysis mapping image was recorded on a FEI TECNAI G2 transmission electron microscope operating at 200 kV. X-ray diffraction (XRD) patterns were collected on a Rigaku D/MAX2550 diffractometer. X-ray photoelectron spectroscopy (XPS) measurements were carried out on a Thermo VG ESCALAB 250 spectrometer. Thermogravimetric analyses (TGA) were taken on a Perkin Elmer Pyris 1 TGA analyzer under N_2_ atmosphere.

### 2.3. Synthesis of BSA-capped Au NCs with red emission

BSA capped Au NCs were prepared using the previously reported procedure.^5^ In a typical experiment, HAuCl_4_ solution (30 mL, 10 mM) was mixed with BSA solution (30 mL, 50 mg mL^−1^) under vigorous stirring at 37 °C. After 2 min, NaOH solution (3 mL, 1 M) was added into the mixture and the solution was kept at 37 °C under vigorous stirring for 12 h. The final solution was deep brown in colour and emitted red fluorescence under 365 nm UV lamp and stored at 4 °C before use.

### 2.4. Synthesis of 11-MUA-capped Au NCs with green emission

11-MUA-capped Au NCs (11-MUA-Au NCs) were synthesized by adopting the method reported in literature.^23^ Briefly, 13.1 mg 11-MUA and NaOH solution (200 μL, 1 M) were mixed with 20 mL water. After complete dissolution of 11-MUA, HAuCl_4_ solution (510 μL, 20 mM) and THPC solution (445 μL, 55 mM) were added. After being kept at 25 °C under vigorous stirring for 2 h, the reaction solution became pale yellow in colour and presented green emission under a 365 nm UV lamp, which was stored at 4 °C before use.

### 2.5. Synthesis of GSH-capped Cu NCs with blue emission

To prepare GSH-capped Cu NCs (GSH-Cu NCs) with blue emission,^24^ first GSH solution (2 mL, 0.1 M) was mixed with CuSO_4_ solution (0.2 mL, 0.1 M). Then NaOH solution (8 mL, 1 M) was added into the mixture to activate the reducing ability of GSH. Final volume of the solution was adjusting to 20 mL with ultrapure water and incubated at 90 °C for 9 h under vigorous stirring. After the reaction, the final solution was pale blue in colour and emitted bright blue fluorescence under 365 nm UV lamp and stored at 4 °C before.

### 2.6. Preparation of metal NCs/BaSO_4_ composites

To prepare the composites of metal NCs/BaSO_4_, first 1.22 g BaCl_2_ (5 mmol) was added into 5 mL aqueous dispersion of the NCs (BSA-Au NCs, GSH-Cu NCs or 11-MUA-Au NCs) under stirring. After 5 min, Na_2_SO_4_ solution (5 mL, 1 M) was added quickly into the above solutions. After being kept for 5 min under stirring, the precipitations were collected by centrifugation (8000 rpm, 5 min) and washed three times by water to get the solid-state metal NCs/BaSO_4_ composites.

### 2.7. Fabrication of light-emitting devices and anti-counterfeiting labels

The metal NCs/BaSO_4_ composites with blue, green and red emissions were dried under vacuum for 48 h at 25 °C and then the solids were ground into fine powders. 0.1 g of the fine powder was mixed with 0.1 g of thermally-curable silicone resin OE-6630A and dried at 50 °C for 1 h. After being mixed with 0.2 g of the OE-6630B hardener, the mixtures were dropped onto the GaN LED chips with the emission wavelength centred at 365 nm to get the corresponding blue, green and red LEDs. To fabricate the white LEDs, first different amount of the NCs with green emission were mixed with the NCs with red emission. When the weight ratio of the green and red NCs was increased to 40 : 1, the mixture became yellow in color. Subsequently, different amount of the NCs with blue emission were mixed with the yellow powder. The final mixture obtained by mixing the metal NCs/BaSO_4_ composites with blue, green and red emissions at an optimal weight ratio of 6 : 40 : 1 presented a white color under a 365 nm UV lamp, which was ground and used to fabricate the white LEDs according to the above procedures. To obtain the anti-counterfeiting labels, 0.1 g of the metal NCs/BaSO_4_ composites with red, green or blue emission was uniformly mixed with 50 μL water and the dispersions were dropped onto stainless steel plates to form the letters “JLU” (the abbreviation of Jilin University) and dried in vacuum at 25 °C for 8 h.

## 3. Results and discussion

**Fig. 1:**
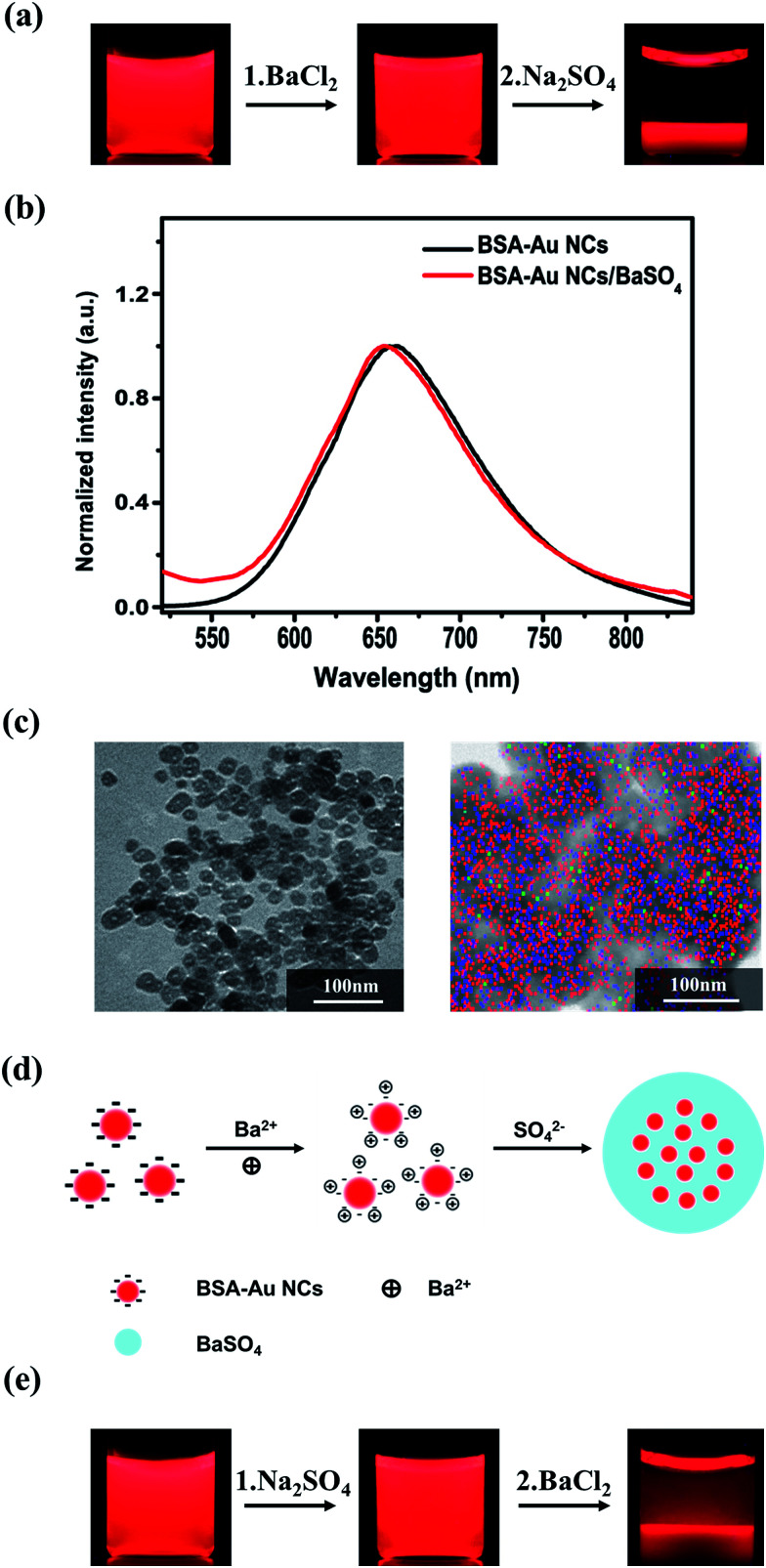
(a) Photos of the dispersion of the as-prepared BSA-Au NCs (left), and the dispersion after the subsequent additions of BaCl_2_ (middle) and Na_2_SO_4_ (right) taken under a 365 nm UV lamp. (b) Emission spectra of the dispersions of the BSA-Au NCs (black curve) and the Au NCs/BaSO_4_ precipitate (red curve). The excitation wavelength was at 365 nm. (c) TEM image (left) and elemental mapping image (right, in which the green, red and blue colours correspond to Au, Ba and S atoms respectively) of the Au NCs/BaSO_4_ composite. (d) Schematic representation for preparation of the Au NCs/BaSO_4_ composite. (e) Photos of the dispersion of the as-prepared BSA-Au NCs before (left) and after the subsequent additions of Na_2_SO_4_ (middle) and BaCl_2_ (right) taken under a 365 nm UV lamp.

The dispersion of BSA-capped Au NCs (BSA-Au NCs) emitted a red emission at 655 nm under 365 nm UV lamp (Fig. 1a, left and Fig. 1b), contributed to the NCs composed of ∼25 Au atoms.^25^ After the addition of BaCl_2_, there was almost no change in emission colour of the dispersion (Fig. 1a, middle). Upon the addition of Na_2_SO_4_, precipitate with red fluorescence was observed and the supernatant become colourless after 5 min (Fig. 1a, right), indicating the complete precipitation of the Au NCs by BaSO_4_. Emission of the BSA-Au NCs presented a slight blue shift (from 655 to 652 nm, Fig. 1b) after being incorporated into the BaSO_4_ matrix, attributed to the change of the surrounding media in the precipitate.^26^ TEM observations show that the precipitate was composed of ellipsoidal particles with an average size of 29 nm (Fig. 1c, left). Mapping images (Fig. 1c, right) confirm the Au NCs were well dispersed in the BaSO_4_ matrix, which is benefit for them to avoid the self-quenching.^17^ XRD pattern of the composite indicates the BaSO_4_ matrix is orthorhombic in structure and the characteristic peaks of BAS-Au NCs^27^ is not observable in the pattern (Fig. S1†) since they are overlapped with those of the orthorhombic BaSO_4_. Strong peak of Ba 3d and S 2p are observed in XPS spectra of the composite, while no peak corresponding to Au 4f was identified (Fig. S2†), suggesting the core–shell like structure of the composite particles, in which the shell is primarily composed by the BaSO_4_ matrix. Zeta-potential measurements indicated that surface charge of the BSA-capped Au NCs was −32 mV, which increased to +4 mV after the addition of BaCl_2_, and then decreased to −2.7 mV after the addition of Na_2_SO_4_, suggesting there are no other functional groups on the surface of Au NCs/BaSO_4_ composite except some adsorbed SO_4_^2−^ ions.

Based on above results, the procedure to prepare the Au NCs/BaSO_4_ composite is illustrated as shown in Fig. 1d. The BSA-Au NCs are negatively charged in the dispersion due to the existence of plenty of carboxyl and hydroxyl groups on BSA capped on surface of the Au NCs (left). After the addition of BaCl_2_, surface charge of the BSA-Au NCs is converted from negative to positive due to the adsorption of Ba^2+^ ions (middle). Emission intensity of BSA-Au NCs changed slightly upon the addition of BaCl_2_ with different concentrations which means that the adsorption of Ba^2+^ ions have little effect on the emission intensity of BSA-Au NCs (Fig. S3†). Upon the subsequent addition of Na_2_SO_4_ solution, rapid precipitation of BaSO_4_ on surface of the Au NCs contributed to formation of the Au NCs/BaSO_4_ composite (right). Control experiments were carried out to further understand the effect of Ba^2+^ adsorbed on the Au NC surface on formation of the Au NCs/BaSO_4_ composite. When Na_2_SO_4_ was first added into the Au NC dispersion, red colour was still observable for the supernatant upon the subsequent addition of BaCl_2_ (Fig. 1e and S4†). This is reasonable since BaSO_4_ precipitate may undergo “self-nucleation” in solution, and become less effective to incorporate the Au NCs. The pre-adsorption of Ba^2+^ ions on the NC surface is necessary to incorporate the Au NCs effectively into BaSO_4_ matrix and direct the formation of Au NCs/BaSO_4_ composite.

**Fig. 2:**
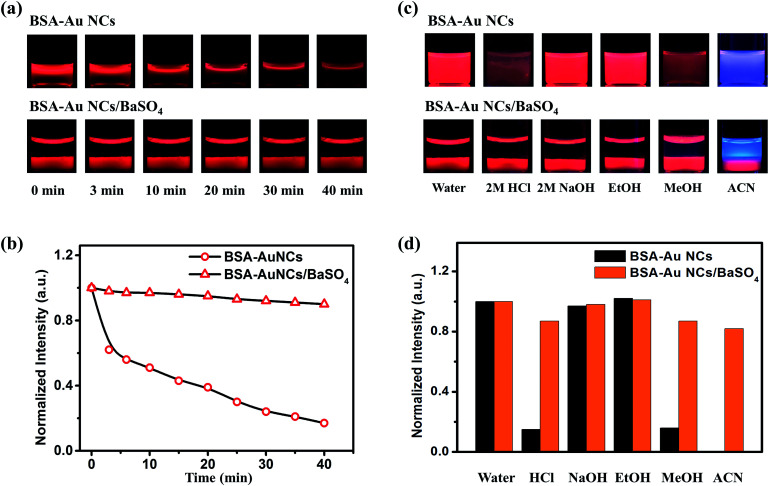
(a) Photos of the BSA-Au NCs (upper panel) and BSA-Au NCs/BaSO_4_ composite (lower panel) after being heated at 90 °C for different time taken under a 365 nm UV lamp. (b) Variation in emission intensity of the BSA-Au NCs at 655 nm and BSA-Au NCs/BaSO_4_ composite at 652 nm with time during the heating process. (c) Photos of the BSA-Au NCs (upper panel) and BSA-Au NCs/BaSO_4_ composite (lower panel) dispersed in different media taken under a 365 nm UV lamp. (d) Comparison of the emission intensity of the BSA-Au NCs at 655 nm and BSA-Au NCs/BaSO_4_ composite at 652 nm after being dispersed in the different media.

According to the procedure shown in Fig. 1d, the preparation of the Au NCs/BaSO_4_ composite could be completed in 10 min at room temperature without using any extra template or organic solvent. Red emission of the BSA-Au NCs was weakened greatly when the solution was heated at 90 °C (Fig. 2a, upper panel), only 18% of the original emission remained after 40 min (Fig. 2b). In comparison, colour of the BSA-Au NCs in the composite was only weakened slightly under the same heating condition (Fig. 2a, lower panel), about 90% of the emission remained after 40 min (Fig. 2b), attributed to the protection effect of the BaSO_4_ matrix. TGA analyses indicated that the pure BSA-Au NCs presented a weight loss of 4.25% at 100 °C, primarily attributed to the loss of BSA. In comparison, only a weight loss of 0.01% of was identified for the Au NCs/BaSO_4_ composite at the same temperature, corresponding to a weight loss of 0.31% for the BSA-Au NCs (Fig. S5†), indicating the improved thermal stability of the BSA-Au NCs in the BaSO_4_ matrix. Therefore the red surface sensitive emission^6^ of the BSA-Au NCs was well kept during the heating process. Further evaluation (Fig. 2c) indicated that both the BSA-Au NCs and the Au NCs/BaSO_4_ composite were stable in neutral and basic aqueous solutions as well as in organic media such as ethanol (>95% remained, Fig. 2d). Although emission of the NCs was quenched obviously in acidic aqueous solution and organic media such as methanol and acetonitrile (<15% remained, Fig. 2d) attributed to the structural change of BSA,^28^ they became more stable after being incorporated into BaSO_4_ (>75% remained, Fig. 2d). It was noted that the Au NCs presented blue violet colour upon being dispersed in acetonitrile, which was blended by the blue emission of acetonitrile and weakened red emission of the Au NCs (<15% remained).

**Fig. 3:**
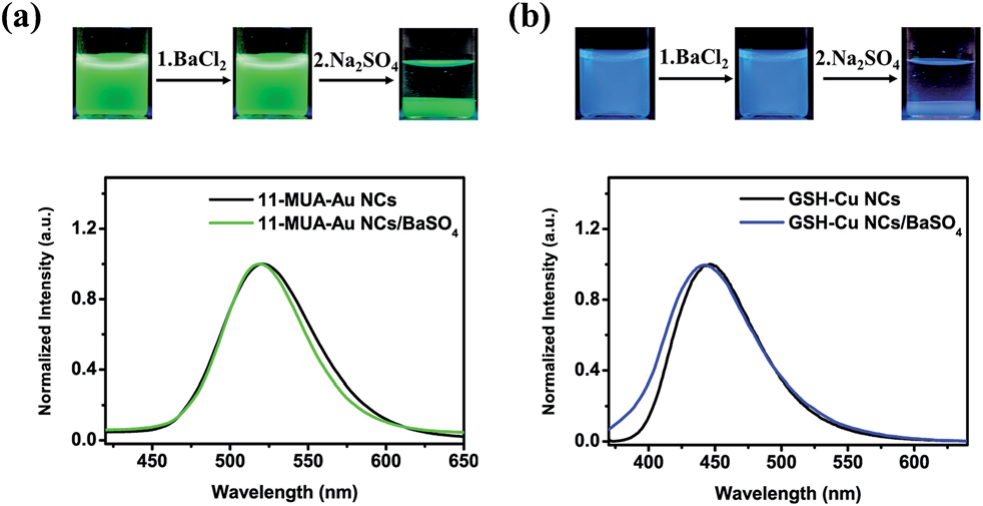
Photos (upper panel) and emission spectra (lower panel) of the (a) 11-MUA-Cu NCs and (b) GSH-Cu NCs before and after the subsequent addition of BaCl_2_ and Na_2_SO_4_. The photos were taken under a 365 nm UV lamp and the spectra were recorded at the excitation wavelength of 365 nm.

The procedure shown in Fig. 1d was extendable to incorporation of other negatively charged metal NCs capped by small organic or biomolecules. For instance, 11-mercaptoundecanoic acid-capped Au NCs (11-MUA-Au NCs, with a zeta-potential value of −29 mV) with green emission at 540 nm attributed to the NCs composed of ∼13 Au atoms^25^ and glutathione (GSH) capped Cu NCs (GSH-Cu NCs, with a zeta-potential value of −22 mV) with blue emission at 445 nm attributed to the NCs composed of ∼15 Cu atoms^29^ could be incorporated effectively into BaSO_4_ matrix by subsequent addition of BaCl_2_ and Na_2_SO_4_ to get the 11-MUA-Au NCs/BaSO_4_ (Fig. 3a, upper panel) and GSH-Cu NCs/BaSO_4_ composites (Fig. 3b, upper panel). The obtained NCs/BaSO_4_ composites maintained the original emission colours of the NCs (Fig. 3, lower panel), suggesting similar to the BSA-Au NCs, the 11-MUA-Au NCs and GSH-Cu NCs could also be well dispersed in the BaSO_4_ matrix.

**Fig. 4:**
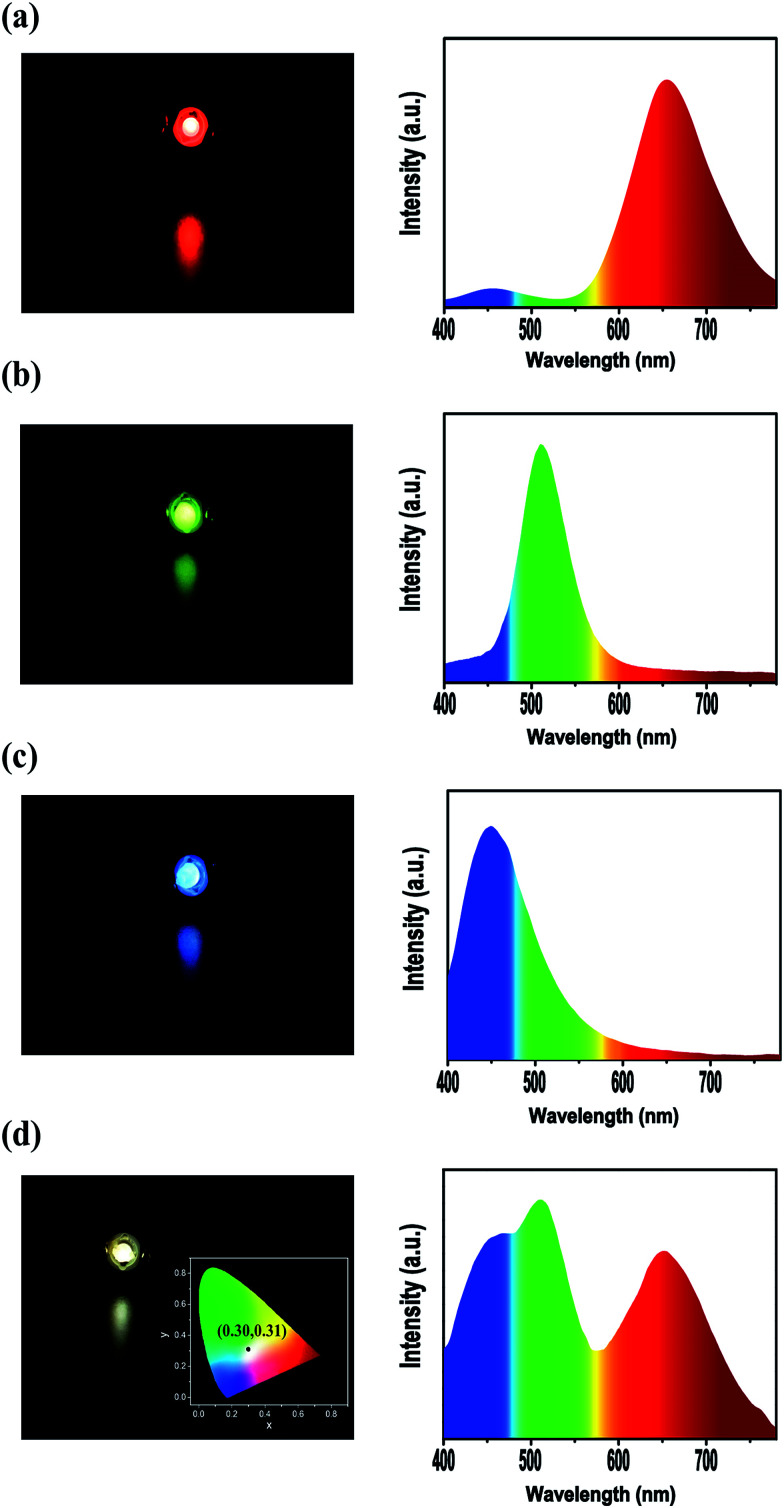
Photos (left) and emission spectra (right) of the working LEDs fabricated by using the (a) BSA-Au NCs/BaSO_4_ composite, (b) 11-MUA-Au NCs/BaSO_4_ composite, (c) GSH-Cu NCs/BaSO_4_ composite and (d) the mixture (6 : 40 : 1 in weight ratio) of the three composites as the monochrome conversion layer. Inset of (d, left) gives the CIE chromaticity coordinates of the corresponding white LED.

The metal NCs/BaSO_4_ composites maintained the emission colours of the original NCs and their dried powders could be readily collected by centrifugation and then vacuum drying. Due to the high reflectivity of BaSO_4_ matrix^30^ and the improved stability of the NCs, the composites were expected to be qualified for device fabrication. Fig. 4 gives the photos and spectra of the LED devices fabricated by using the dried powders of the composites as the colour conversion layers deposited on commercial UV GaN LED chips. Colours and emission spectra of the red, green and blue LED devices (Fig. 4a–c) well matched those of the original metal NCs, meaning BaSO_4_ is an ideal matrix for incorporating the luminescent metal NCs. Interestingly, the LED devices based on the mixed powders containing an optimal weight ratio (6 : 40 : 1) of the blue, green and red NCs presented a white colour with an emission spectrum covering the entire visible region (400–700 nm, Fig. 4d), with the CIE chromaticity coordinates (inset of Fig. 4d) of (0.30, 0.31) close to the ideal value for pure white emission (0.33, 0.33). Departure of the weight ratio from the optimal value will produce LEDs less pure in white color. For example, the LEDs become blue white in color if excess amount of the blue NCs was added and yellow white in color when the amount of the blue NCs was insufficient.

**Fig. 5:**
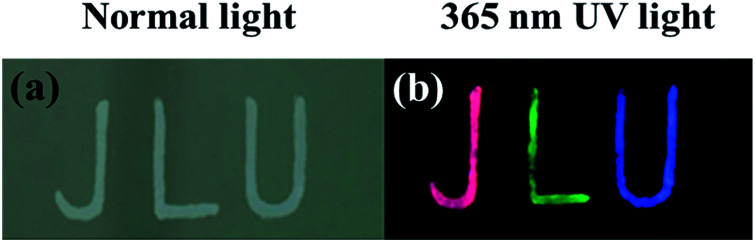
Photos of the patterns fabricated by the BSA-Au NCs/BaSO_4_ (J), 11-MUA-Au NCs/BaSO_4_ (L) and GSH-Cu NCs/BaSO_4_ composites (U) written on stainless steel plates taken under normal light (a) and 365 nm UV lamp (b).

The letters “JLU” (abbreviation of Jilin University) obtained by dropping the different composites onto stainless steel plates are all white in colour under normal light (Fig. 5a). Under a 365 nm UV lamp, the letters “J”, “L” and “U” presented red, green and blue colours respectively, dependent on kinds of the composites used to fabricate the patterns (Fig. 5b). This feature gives the opportunity to fabricate anti-counterfeiting patterns from the composites. Compared with other luminescent materials,^31,32^ the NCs/BaSO_4_ composites are more eco-friendly, less toxic, and more stable, making them ideal luminescent materials for fabrication of devices and anti-counterfeiting patterns.

## 4. Conclusions

In summary, we reported the facile incorporation of luminescent metal NCs with red, green and blue emission colours into BaSO_4_ matrix. Preparation of the composites was completable in 10 min at room temperature without the requirement of any extra template or organic solvent. The resulting metal NCs/BaSO_4_ composites maintained the luminescent colours of the original metal NCs and their stability was improved greatly compared to the original NCs. The solid-state composites were qualified for fabrication of white LEDs and multicolour anti-counterfeiting labels. Such metal NCs-based monolithic materials are expected to hold great promise in information security and lighting technology.

## Conflicts of interest

There are no conflicts to declare.

## Supplementary Material

RA-008-C7RA11804J-s001
