# Supplementary material for: Luminescent metal clusters/barium sulfate composites for white light-emitting devices and anti-counterfeiting labels
Source: RSC Adv. 2018 Jan 15;8(6):2866–71. doi: 10.1039/c7ra11804j (PMC9077558; doi:10.1039/c7ra11804j)
Supplement: RA-008-C7RA11804J-s001 [file RA-008-C7RA11804J-s001.pdf]

Electronic Supplementary Material (ESI) for RSC Advances.
This journal is © The Royal Society of Chemistry 2018

Electronic Supplementary Information

Luminescent metal clusters/barium sulfate composites for white light-emitting devices and anti-counterfeiting labels

Min Wang, Zhenzhen Huang, Zilong Guo and Wensheng Yang*

E-mail: wsyang@jlu.edu.cn

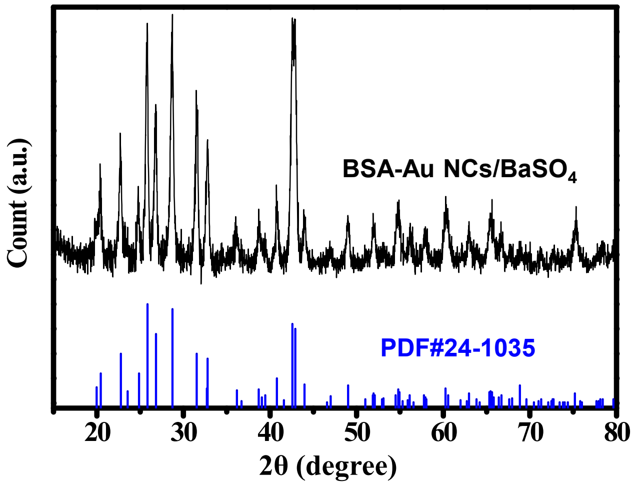

Fig. S1. XRD pattern of the powdered BSA-Au NCs/$BaSO_4$ composite. The peaks are well fitted to the standard pattern of orthorhombic $BaSO_4$ (blue). No characteristic diffraction peaks of the BSA-Au NCs at 31.82°, 45.65° and 63.70° corresponding to (111), (200) and (220) planes of face centered cubic gold were observed since they are rather weak and overlapped with those of the $BaSO_4$ matrix.

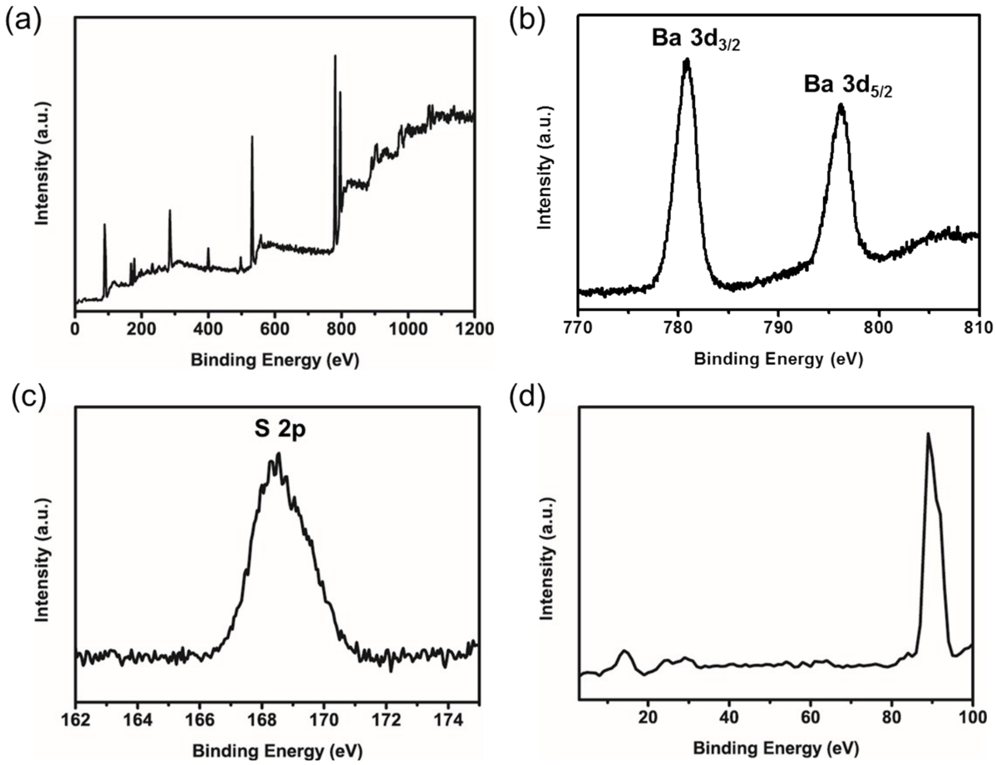

Fig. S2. XPS spectra of the BSA-Au NCs/$BaSO_4$ composites in the region of (a) 0−1200 eV, (b) 770−810 eV, (c) 162−175 eV and (d) 0−100 eV. The peaks of Ba 3d3/2 and 3d5/2 and S 2p could be well identified in the spectra. However, the peaks of Au 4f5/2 (ca. 87.7 eV) and 4f7/2 (ca. 84.0 eV) were not observed. These results mean that the BSA-Au NCs were mainly located in the $BaSO_4$ matrix but not on surface of the composite particles, suggesting the core-shell like structure of the particles.

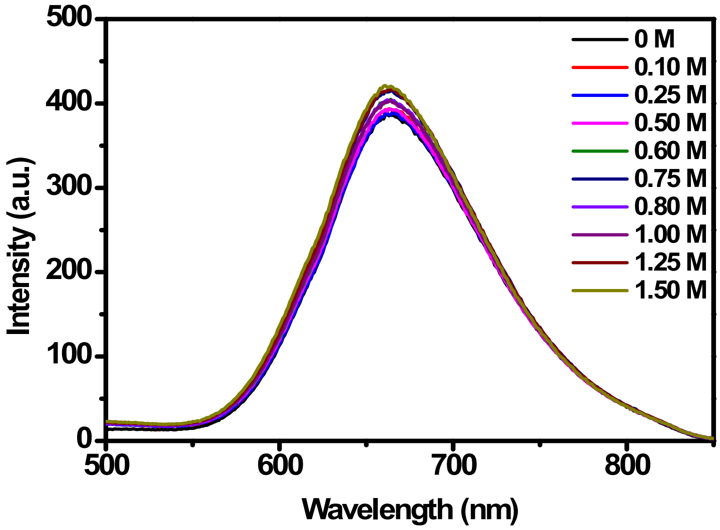

Fig. S3. Emission spectra of the BSA-Au NCs dispersed in $BaCl_2$ solutions with different concentrations. Emission intensity of the BSA-Au NCs changed slightly with the concentrations of $BaCl_2$, meaning the adsorption of Ba^{2+} ions have little effect on the emission intensity of the BSA-Au NCs.

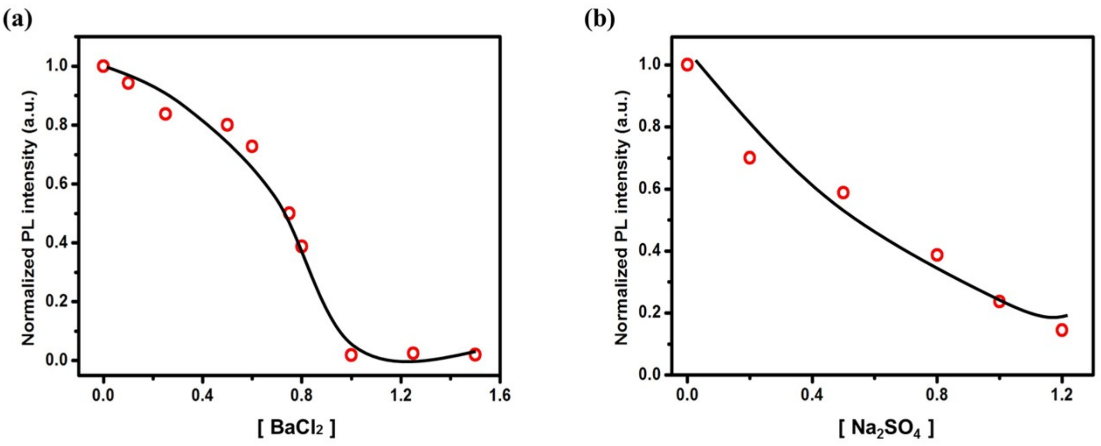

Fig. S4. Variation in emission intensity of the supernatants after centrifugation separation of the BSA-Au NCs/$BaSO_4$ composites prepared by altering the adding sequences of $BaCl_2$ and Na_2SO_4. (a) First addition of $BaCl_2$, and then subsequent addition of equal molar Na_2SO_4. When the concentration of $BaCl_2$ is more than 1 M, no red color was observed in the supernatants, indicating the complete encapsulation of the BSA-Au NCs into the $BaSO_4$ matrix. Thus, the molar concentrate of $BaCl_2$ was selected to be 1 M to prepare the composites. (b) First addition of Na_2SO_4, and then subsequent addition of equal molar $BaCl_2$. Red color of the BSA-Au NCs was still observable when the concentration of Na_2SO_4 was higher than 1.2 M.

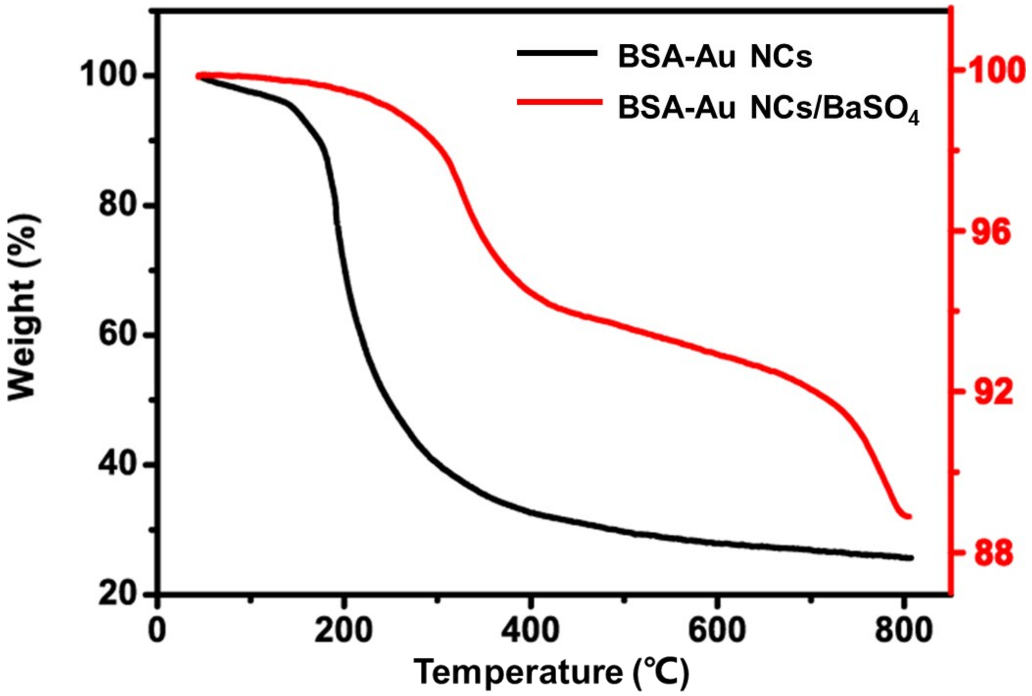

Fig. S5. TGA thermograms of the BSA-Au NCs (black curve) and the BSA-Au NCs/$BaSO_4$ composite (red curve). According to the experimental section, when the concentration of $BaCl_2$ is 1 M (5 ml, corresponding to 1.1670 g $BaSO_4$), the NCs could be completely incorporated into $BaSO_4$ matrix. After centrifugation, weights of dried powders of the BSA-Au NCs/$BaSO_4$, 11-MUA-Au NCs/$BaSO_4$ and GSH-Cu NCs/$BaSO_4$ composites were 1.2057, 1.1950 and 1.1854 g, respectively. After subtraction of the weight of $BaSO_4$, the weights of the BSA-Au NCs, 11-MUA-Au NCs and GSH-Cu NCs were derived to be 38.7, 28.0 and 18.4 mg, corresponding to weight ratios of 3.21%, 2.34% and 1.55% respectively. A weight loss of 4.25 % was identified for the pure BSA-Au NCs when the temperature exceeded 100 ℃ and only a weight loss of 0.01% was identified for the BSA-Au NCs/$BaSO_4$ composite at the same temperature, corresponding to a weight of 0.31% for the BSA-Au NCs, indicating the improved thermal stability of the BSA-Au NCs in the composite.
